# Bacteriophage-Enriched Galenic for Intrapericardial Ventricular Assist Device Infection

**DOI:** 10.3390/antibiotics11050602

**Published:** 2022-04-29

**Authors:** Sebastian V. Rojas, Simon Junghans, Henrik Fox, Kanstantsin Lazouski, Rene Schramm, Michiel Morshuis, Jan F. Gummert, Justus Gross

**Affiliations:** 1 Clinic for Thoracic and Cardiovascular Surgery, Heart and Diabetes Centre North Rhine Westphalia, University Hospital, Ruhr-University Bochum, 32545 Bad Oeynhausen, Germany; hfox@hdz-nrw.de (H.F.); klazouski@hdz-nrw.de (K.L.); rschramm@hdz-nrw.de (R.S.); mmorshuis@hdz-nrw.de (M.M.); jgummert@hdz-nrw.de (J.F.G.); 2 G. Pohl-Boskamp GmbH & Co. KG, 25551 Hohenlockstedt, Germany; s-junghans@web.de; 3 Department for General, Visceral, Vascular and Transplantation Surgery, Rostock University Medical Center, 18057 Rostock, Germany; justus.gross@med.uni-rostock.de

**Keywords:** LVAD, left ventricular assist device, infections, bacteria, bacteriophages, negative pressure wound therapy, NPWT

## Abstract

We report a case of severe outflow graft infection following left ventricular assist device (LVAD) implantation. A 51-year old male LVAD patient was readmitted to our hospital presenting signs of systemic infection. One year previously, LVAD implantation (HeartMate3, Abbott, Chicago, IL, USA) with concomitant patent foramen ovale closure had been performed in the context of end-stage heart failure due to dilative cardiomyopathy (INTERMACS III). The indication for LVAD-therapy was bridge-to-candidacy, since the patient did not instantly fulfill all criteria for cardiac transplantation. At admission, a PET-CT scan unveiled fluid accumulation, encircling the outflow-graft prosthesis (SUV_max_ 10.5) with contrast-enhancement involving the intrathoracic driveline (SUV_max_ 11.2). Since cardiac transplantation was not feasible, the patient underwent surgical revision. In the first step, redo sternotomy was performed with local debridement, including jet lavage. Intraoperative swabs confirmed bacterial infection with *staphylococcus aureus*. Following this, the patient underwent negative pressure wound therapy (NPWT) with instillation using the V.A.C. VERAFLO system (KCI-3M, San Antonio, TX, USA) for a total of 19 days. Due to the severity of infection, local bacteriophage application was performed within the wound closure. In order to concentrate phage therapy at the infection site, phages were applied using a novel semi-fluid galenic. After wound closure, the patient was discharged with an uneventful course. A control PET-CT scan 3 months after discharge showed a significant decrease in infection (outflow graft: SUV_max_ 7.2, intrathoracic driveline: SUV_max_ 3.0) correlated with contrast enhancement. Bacterial infection of intrathoracic VAD components represents a severe and potentially life-threatening complication. If cardiac transplantation is not feasible, complex wound management strategies are required. Local bacteriophage therapy might be a promising addition to already established therapeutical options. In order to improve bacteriophage retention at the wound site, application of a viscous galenic might be beneficial.

## 1. Introduction

Despite significant advances over the past two decades in the field of long-term mechanical circulatory support, infections have remained an unsolved subject since the establishment of ventricular assist device (VAD) therapy [1]. VAD infections are not only a common complication, but also an important risk factor for mortality and morbidity, limiting therapy success [2]. Depending on the definition and extent of infection, reported incidences vary between 13 and 80%. VAD infections might be limited to the driveline exit site, but can also involve internal components such as the pump housing or the outflow graft [3]. In these cases, cardiac transplantation is the best strategy. This way, all infectious extraneous material is eliminated entirely. However, donor organ availability is strongly limited, and there is a growing number of destination therapy patients who will require alternative treatment in case of device infection [4]. This is particularly important, as otherwise established strategies such as antibiotic treatment or negative pressure wound therapy alone have not been able to provide an effective solution for device-related infections [5]. Lately, the rediscovery of bacteriophage therapy for the treatment of complex surgical infections has enabled a stimulating and novel, yet poorly investigated, option for VAD patients with device-related infections [6,7,8,9].

Herein, we report a case of severe infection of an outflow graft which was successfully treated by applying complex wound management, including local bacteriophage therapy at wound closure.

## 2. Medical History

**Figure 1:**
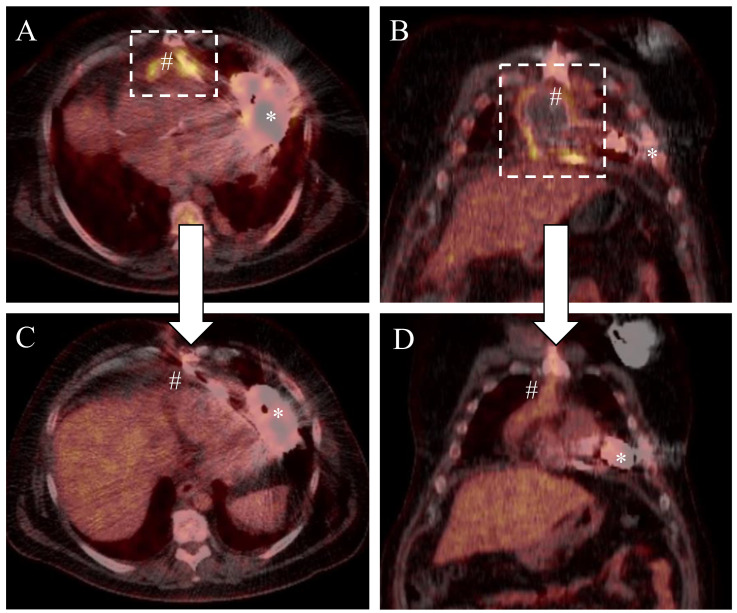
PET-CT scan before (**A**,**B**) and after (**C**,**D**) complex wound treatment. # Outflow graft of the LVAD-System; * Pump housing of the LVAD (HeartMate 3, Abbott, USA). The dashed rectangle shows contrast enhancement involving the outflow graft as a correlate for device infection.

A 49-year-old male patient with a history of dilative cardiomyopathy and end-stage heart failure (NYHA IV, INTERMACS III) underwent LVAD implantation (HeartMate 3, Abbott) and concomitant patent foramen ovale closure in our institution. Indication for LVAD therapy was bridge-to-candidacy, as the patient was additionally suffering from untreated reactive depression. Risk factors for infection were the INTERMACS status, overweight (BMI 34 kg/m^2^) and insulin-dependent diabetes (Type II). After an uneventful intrahospital clinical course for LVAD implantation, the patient was discharged. However, 6 months after LVAD implantation, the patient was readmitted to the ICU presenting dyspnea, systemic infection signs and left-sided chest pain. Computer tomography unveiled a thoracic empyema and consequently a left thoracotomy was performed for drainage and pleural decortication. Intraoperative bacterial swabs confirmed the presence of *staphylococcus aureus* (penicillin R > 0.5; levofloxacin I < 0.12). Therefore, in addition to surgery, guided antibiotic therapy (fosfomycin and floxacillin) was initiated. The patient recovered well and could be discharged without antibiotic therapy one month after decortication. Six months later, the patient was again re-admitted to our institution presenting with septicemia with *staphylococcus aureus*. An integrated positron emission tomography/computed tomography (PET/CT) scan (Biograph mCT, Siemens Healthineers AG, Erlangen, Germany) with the glucose analogue 2-(18)F-fluoro-2-deoxy-d-glucose (FDG) (Figure 1A,B) unveiled two intrathoracic areas of activity enrichment, one surrounding the outflow graft (SUV_max_ 10.5) and another involving the intrathoracic driveline (SUV_max_ 11.2). Thus, complex surgical wound management including bacteriophage application was planned for the treatment of this severe infection. Patient consent was obtained for an individual bacteriophage treatment respecting the ethical principles for medical research of the Helsinki declaration.

## 3. Surgical Wound Management and Postoperative Outcome

**Figure 2:**
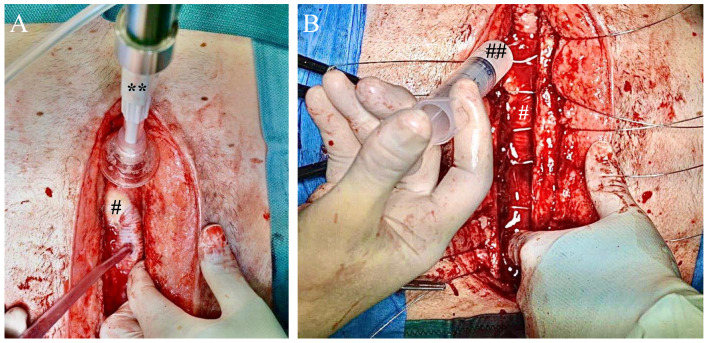
Intraoperative setup during wound closure. **(A**) For debridement purposes, pulsatile jet lavage (**) was applied in order to minimize local bacterial concentrations at the outflow graft (#). (**B**) Viscous bacteriophage-enriched galenic (##) was injected at the infection site before sternal closure with standard stainless-steel cerclages.

In the first step, redo sternotomy was performed. Intraoperatively, bacterial infection was confirmed. In order to reduce the bacterial load, local wound debridement including pulsating jet lavage (PALAVAGE, Heraeus, Wehrheim, Germany) was performed with antiseptic (LAVANOX, Serag-Wiesner, Naila, Germany) and isotonic solution (Figure 2A). Furthermore, a combination of negative-pressure wound therapy (NPWT) coupled with automated controlled instillation therapy was performed for a total of 19 days using the V.A.C. VERAFLO system (KCI-3M, USA). As an instillation solution we used LAVANOX (Serag-Wiesner, Naila, Germany), which was instilled for 10 min every 4 h. NPWT was conducted at 75 mmHg. Due to the complex wound geometry, a tubular-shaped V.A.C. Veraflo Cleanse (KCI-3M, USA) was selected as the wound dressing and was adapted to fill the entire wound. It was exchanged regularly according to the product specifications. After 19 days and confirmation of a positive wound-healing tendency, definite wound closure was performed. For debridement purposes, pulsating jet lavage was performed again, applying antiseptic and saline solution. Following this, a bacteriophage mixture (SniPha 360, Sanubiom GmbH, Fritzens, Austria) (20 mL, concentration 1 × 10^7^ CFU/mL) with bacteriophages against Staphylococcus aureus, Escherichia coli, Pseudomonas aeruginosa, Streptococcus pyogenes, Enterococcus faecalis, Proteus vulgaris and Proteus mirabilis, was administered using a novel viscous galenic as the injection media. The pharmaceutical requirements for the galenic dosage form were based on the conventional three pharmaceutical requirements: quality, safety and efficacy. To maintain bacteriophage efficacy, the composition was chosen to ensure that the phages would remain lytic and thereby therapeutically active in the viscous matrix. Safety was ensured by making toxicological considerations and via the use of components with well-established routines in pharmaceuticals and showing no interactions or other side effects in vitro. The overall quality of the composition resulted from the individual substance qualities of the components used as well as a document-based audit of the phage supplier, which ensured that the phages applied meet the GMP requirements.

Medical requirements of the galenic were based on the patient’s wound situation. Due to the deep infection, fluidity had to be ensured for an encompassing coverage of the infective tissue. In addition, a modified release galenic was needed, that would release the phages over a predetermined period of time, tested in vitro, so that not only acute but prolonged antibacterial phage therapy would be achieved after application. The medical and pharmaceutical requirements were implemented in a viscous, aqueous product that primarily contained a pH buffer to maintain neutral pH under an inflammatory and acidic environment and also phage stabilizing components to ensure the prolonged effectiveness of the phage therapy after application.

Sternal wound closure was performed as usual using stainless-steel cerclage wire for bone fixation and absorbable single sutures for the pectoral muscle and subcutaneous tissue. The cutis was closed applying Donati’s suture technique. After an uneventful period of 11 days and further antibiotic therapy (piperacillin/tazobactam 4.5 g, 3×/day), the patient was successfully discharged from hospital. After 3 months, a PET-CT scan (Figure 1C,D) was performed in order to corroborate therapy success. Imaging unveiled a significant decrease in contrast enhancement (outflow graft: SUV_max_ 7.2, intrathoracic driveline: SUV_max_ 3.0), suggesting a mitigation of the outflow graft infection. Six months later, the patient was readmitted to our hospital showing a local infection of the driveline exit site with *Stapylococcus aureus* (Penicillin R > 0.5; Levofloxacin I < 0.12), but without interrelation to the former intrathoracic focus. Since the patient did not show signs of a systemic infection, conservative therapy (local treatment with disinfectants and wound dressing changes) without systemic antibiotic was initiated.

## 4. Discussion

A growing number of antibiotic-resistant bacterial strains and the increase in cardiovascular implants has generated the need for alternative treatment strategies in implant-associated infections [10]. Especially in VAD-therapy, pump-associated infections represent an important and therapy-limiting factor in the long-term support of mechanical circulatory [11]. While local driveline infections of the exit site may be treated conservatively, infections of the intrathoracic pump components represent a life-threatening complication. In these patients, LVAD exchange is an alternative, but results have been poor, with an in-hospital mortality of 10% and a recurrence of infection of 60% [12]. Therefore, in these cases, heart transplantation currently remains the most favorable option capable of eradicating the infection focus. However, the availability of donor organs is limited, and a considerable number of LVAD patients do not qualify for transplantation, as they are considered destination therapy (DT) patients.

With the recent rediscovery of bacteriophage therapy, certain reports have described successful treatment of bacterial infections, including LVAD infections [6,13].

In this case report we showcase the successful treatment of a severe bacterial outflow-graft infection within a LVAD patient that could not be enlisted for heart transplantation, using a complex wound management strategy including bacteriophage application. In the first step, debridement of the infected tissue was performed, followed by jet lavage and NWPT with consecutive intermittent instillation of antiseptic solution. In our opinion, the conditioning of the wound is essential for therapy success. To promote wound tissue granulation and to maintain wound flexibility for further closure, we selected a tubular-shaped dressing (V.A.C. VERAFLO, KCI-3M, USA). Furthermore, we continued with intravenous antibiotic therapy. Ultimately, a mixture of bacteriophages effective against different types of bacteria was applied. Despite the fact we only identified Staphylococcus aureus previously, we also wanted to cover other types of bacteria potentially present at the wound site. In the future, distinct combinations comprising different stems of bacteriophages might help to standardize this type of treatment. In general, we also consider it highly essential to standardize assays for gauging bacteriophage effectiveness against specific bacteria in future approaches. In this specific case, we did not perform this form of analysis.

As a suitable injection vehicle, we aimed at finding a medium capable of attaching to the infected tissue and then releasing active bacteriophages over an extended period of time. While others have focused on using fibrin glue for this purpose [10], we decided to apply a hydrophilic galenic that met the prevailing pharmaceutical requirements. Moreover, biocompatibility and in vitro release results were known, and the intended scope of the distribution was determined by the composition.

In summary, we present a novel technique for the treatment of severe intrathoracic LVAD infection. Since this is a single case report with inherent limitations, further studies with larger patient numbers and evaluation of bacteriophage therapy are required. This report is also limited, as direct conclusions regarding the anti-inflammatory effect of the bacteriophage application cannot be distinguished from the effect of the mechanical wound cleaning, antibiotic treatment or NPWT.

## Data Availability

Not applicable.
